# Supplementary material for: The causal role of circulating inflammatory markers in osteoporosis: a bidirectional Mendelian randomized study
Source: Front Immunol. 2024 Jul 18;15:1412298. doi: 10.3389/fimmu.2024.1412298 (PMC11291241; doi:10.3389/fimmu.2024.1412298)
Supplement: Supplementary file 1 [file DataSheet_1.zip › supplementary material/CIM-OSTEOPOROSIS /GCST90274820.csv_forest.pdf]

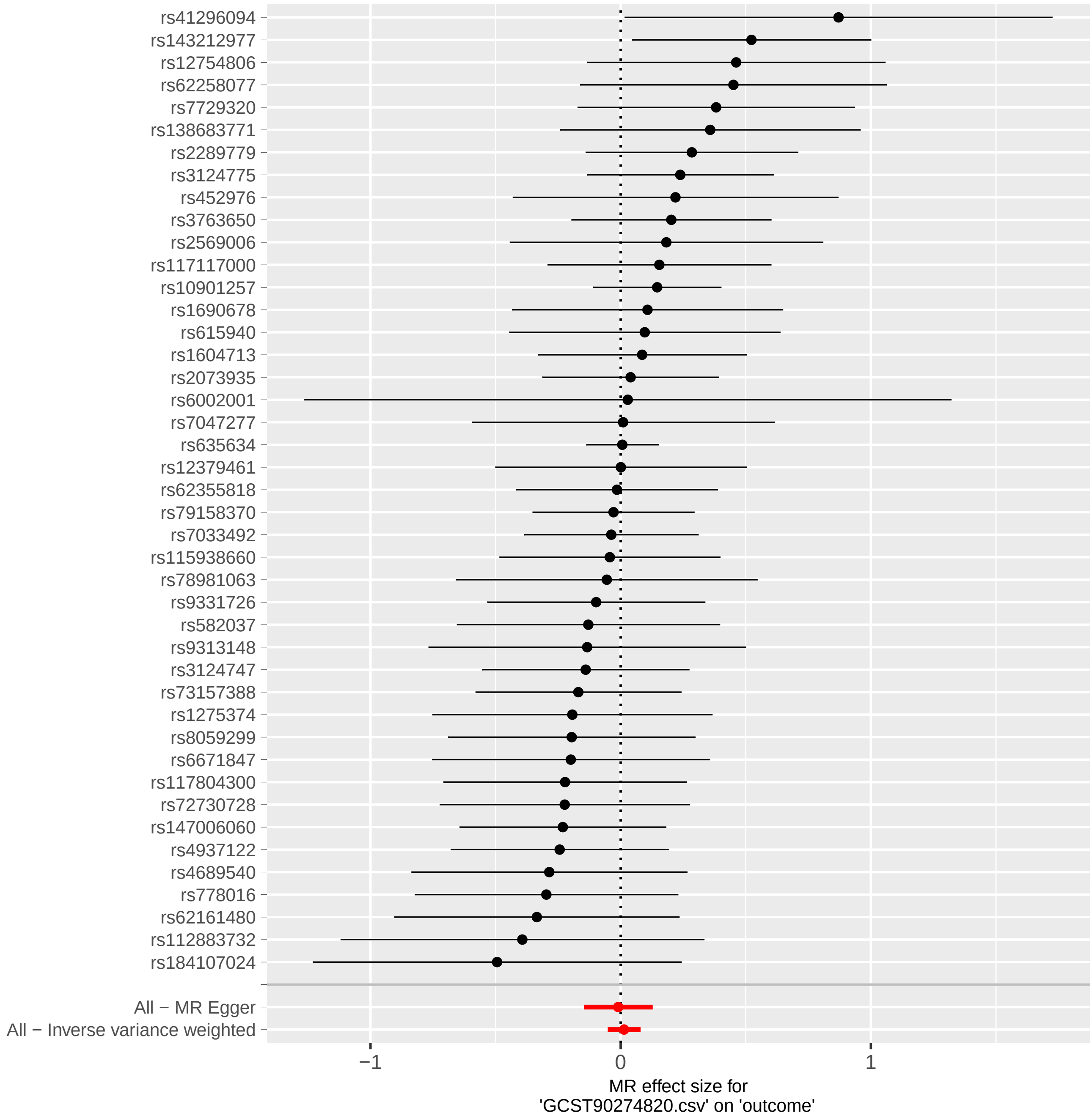

rs41296094
rs143212977
rs12754806
rs62258077
rs7729320
rs138683771
rs2289779
rs3124775
rs452976
rs3763650
rs2569006
rs117117000
rs10901257
rs1690678
rs615940
rs1604713
rs2073935
rs6002001
rs7047277
rs635634
rs12379461
rs62355818
rs79158370
rs7033492
rs115938660
rs78981063
rs9331726
rs582037
rs9313148
rs3124747
rs73157388
rs1275374
rs8059299
rs6671847
rs117804300
rs72730728
rs147006060
rs4937122
rs4689540
rs778016
rs62161480
rs112883732
rs184107024
All − MR Egger
All − Inverse variance weighted
−1
0
1
MR effect size for
'GCST90274820.csv' on 'outcome'
